# Joint damage in rheumatoid arthritis: assessment of a new scoring method

**DOI:** 10.1186/ar4163

**Published:** 2013-02-13

**Authors:** Alexander Pfeil, Peter Oelzner, Klaus Bornholdt, Andreas Hansch, Gabriele Lehmann, Diane M Renz, Gunter Wolf, Joachim Böttcher

**Affiliations:** 1 Department of Internal Medicine III, Jena University Hospital - Friedrich Schiller University Jena, Erlanger Allee 101, 07747 Jena, Germany; 2 Sanofi-Aventis Deutschland GmbH, Potsdamer Straße 8, 10785 Berlin, Germany; 3 Institute of Diagnostic and Interventional Radiology, Jena University Hospital - Friedrich Schiller University Jena, Erlanger Allee 101, 07747 Jena, Germany; 4 Department of Diagnostic Radiology, Charité University Medicine Berlin, Campus Virchow Clinic, Augustenburger Platz 1, 13353 Berlin, Germany; 5 Institute of Diagnostic and Interventional Radiology, SRH Wald-Klinikum Gera, Straße des Friedens 122, 07548 Gera, Germany

## Abstract

**Introduction:**

The aim of this study was to assess a novel approach for the quantification of finger joint space narrowing and joint destruction in patients with rheumatoid arthritis (RA) focusing on the peripheral hand articulations.

**Methods:**

A total of 280 patients with verified RA underwent computerized semi-automated measurements of joint space distance at the finger articulations based on radiographs. The Z-Score, which can differentiate between joint space alterations caused by RA versus age/gender-related changes, was calculated as a comparative parameter. The severity of joint space narrowing was also quantified by the Sharp Score. Sensitivity and specificity of the Z-Score (based on joint space widths differentiated for each peripheral finger joint) were evaluated to reveal the potential for the occurrence of erosions. Additionally, the potential of the Z-Score regarding the differentiation of therapeutic effects on joint space widths in patients under a therapy of methotrexate versus leflunomide was performed.

**Results:**

The Z-Scores of finger articulations in patients with RA were generally decreased. Metacarpal-phalangeal (MCP) joint articulations showed a continuous significant decline of -1.65 ± 0.30 standard deviations dependent on the Sharp Score. The proximal-interphalangeal joints also revealed a significant reduction of the Z-Score (-0.96 ± 0.31 standard deviations). The sensitivity and specificity of MCP joint space distance for the detection of erosions were 85.4% versus 55.2%. The Sharp Score for joint space narrowing was not able to detect different treatments, whereas an accentuated stabilization of joint space narrowing could be identified for the Z-Score of the MCP joints in patients treated with leflunomide and methotrexate.

**Conclusion:**

The Z-Scoring method based on computer-aided analysis of joint space widths was able to reliably quantify severity-dependent joint space narrowing in RA patients. In the future, calculation of a Z-Score based on gender-specific and age-specific reference data shows the potential for a surrogate marker of RA progression that comprehends the early identification of patients with RA, and in particular those with erosive course of the disease, enabling a timely therapeutic strategy for cartilage protection.

## 

Rheumatoid arthritis (RA) is a chronic inflammatory disease characterized by synovial inflammation leading to cartilage destruction and resulting in joint space narrowing, bone erosions, and periarticular demineralization. Consequently, RA scoring from radiographs involves three aspects: bone mineral density, joint space width, and hand erosion count. Besides the measurement of disease activity, a major outcome criterion of clinical trials is the assessment of radiographic progression based on the detection of erosions and joint space narrowing [1]. However, currently established scoring methods, although widely applied, have been associated with several limitations such as limited generalizability and objectivity due to the difficulty of standardized scoring by different readers with variable experience [2].

Recent advances in computer-aided diagnosis now offer the opportunity for a standardized measurement of radiographically visible alterations focusing on the small joints of the hand [3], with a focus on the assessment of joint space widths [4]. Computer-based methods for the measurement of joint space width could provide substantial advantages in comparison with the assessment of joint space narrowing by manual scoring methods, because of improved standardization, sensitivity, and reproducibility [5,6].

Computer-aided joint space analysis (CAJSA) is a relatively new technique that performs semi-automated measurements of joint space distances (JSDs) at the finger articulations using digitized hand radiographs [7]. Recently, new data have shown an age-specific and gender-specific joint space narrowing in healthy subjects and RA patients [7,8].

Pfeil and colleagues introduced the Z-Score to differentiate RA-induced joint space narrowing from age-related and gender-related changes of finger joint space widths [9]. The aim of this study was to assess the potential of this novel approach based on Z-Score calculations to reliably quantify finger joint space narrowing in RA patients as well as to illustrate its sensitivity and specificity depending on the occurrence of bone erosions. Additionally, the clinical relevance of the Z-Score was determined in the comparison of two different patient groups treated with methotrexate and leflunomide considering a head-to-head comparison of manual and automated joint space narrowing scoring.

## Materials and methods

### Patients

The current study consisted of 280 patients (201 women and 79 men) with RA as defined by the American College of Rheumatology 1987 criteria [10]. The study was divided into two parts with a cross-sectional and a longitudinal analysis of the Z-Score using a head-to-head comparison of manual and automated joint space narrowing scoring.

#### Cross-sectional Z-Score analysis

A total of 186 patients (133 female and 53 male) with age ranging from 33.0 to 77.0 years (mean ± standard deviation (SD): 54.7 ± 11.1 years) and mean disease duration of 7.6 ± 8.4 years were included in the first part of the section. No preselection regarding the grade of RA or steroid therapy was considered. All patients were treated with disease-modifying antirheumatic drugs and 91 patients were on prednisolone therapy (mean dosage: 5 mg/day). Patients with signs of fracture or visible osteosynthetic material as well as patients showing one joint with a Sharp Score for joint space narrowing of 4 were excluded.

#### Longitudinal Z-Score analysis

The second part of the study included 94 patients (68 women and 26 men) participating in the LEMERADIX REGISTER (Retrospective Comparison of Leflunomide and Methotrexate in Rheumatoid Arthritis by Digital Radiogrammetry and CAJSA) as a prospectively planned, comparative, multicenter retrospective study in patients suffering RA. The mean age and disease duration was 54.8 ± 13.0 years versus 2.4 ± 5.6 years. Fifty-three patients were treated on average with 15 mg/week methotrexate and 41 patients were treated with leflunomide (10 mg/day, five patients; 20 mg/day, 36 patients). Of the patients, 55% were positive for antibodies to citrullinated proteins and 64% were positive for rheumatoid factor. The time difference between the first and second X-ray scans was 1.87 ± 0.68 years. All patients had to fulfill the following criteria: monotherapy with either leflunomide or methotrexate during the entire documentation period; no combination therapy of leflunomide or methotrexate with other disease-modifying antirheumatic drugs; no intake of bisphosphonates or hormone replacement therapy during the documentation period; an available X-ray scan of one hand at the start of therapy with leflunomide or methotrexate (± 3 months); radiographs of the same hand from the time period 1 to 3 years after the start of therapy with leflunomide or methotrexate; age ≥18 years; and patient informed consent prior to inclusion. Patients with visible osteosynthetic material were excluded.

### Methods

#### Computer-aided joint space analysis for quantifying joint space width

**Figure 1:**
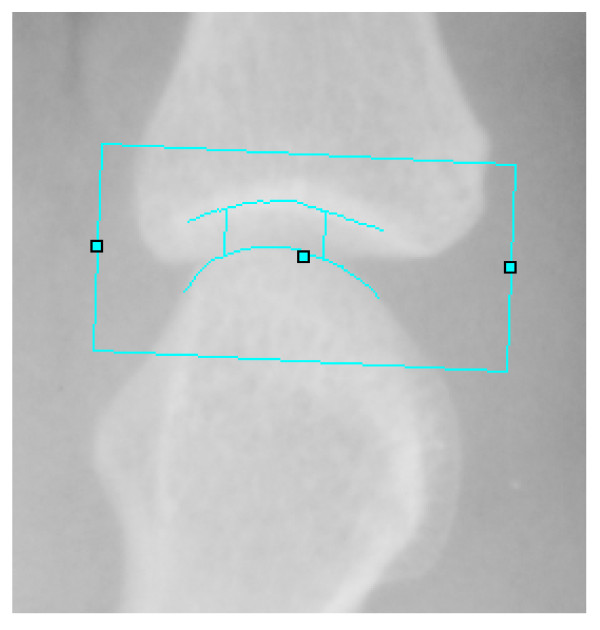
**Hand radiograph illustrating region of interest measurement of joint space width at the metacarpal-phalangeal articulation**. The joint edges were detected as intensity maximum and the joint space distances were measured with 180 measurement points per centimeter between the two lines, which define the cortical layer of the articulation bones.

CAJSA (version 1.3.6; SectraLinköping, Sweden) was used to measure joint space widths of the metacarpal-phalangeal (MCP), proximal-interphalangeal (PIP) and distal-interphalangeal (DIP) joints of each finger of both hands. This technique was introduced as a semi-automated measurement of finger joint space widths and has been described in detail by Pfeil and colleagues [7]. The region of interest for measurement of the joint space width was located by the operator as a unique user-dependent procedure during the measurement process. Subsequently, the CAJSA technique automatically measures JSDs (see Figure 1), obtaining excellent reproducibility [11]. Additionally, the region of interest was not located in joint areas with erosive destructions of the cortical layer to prevent reliable estimates. The time required for analysis of one joint is less than 90 seconds. To quantify age-independent and gender-independent joint space narrowing, the Z-Score of the CAJSA measurements was used [9]. The Z-Score is calculated for each joint as follows and is expressed in SDs:

$$\text{Z - Score}=\left(\text{JS}{\text{D}}_{\text{patient}}-\text{JS}{\text{D}}_{\text{age}-\text{matched}\;\text{and}\;\text{gender}-\text{matched}\;\text{control}}\right)\%\text{S}{\text{D}}_{\text{age}-\text{matched}\;\text{and}\;\text{gender}-\text{matched}\;\text{control}}$$

The used reference collective were characterized as published by Pfeil and colleagues [7]. For the reference collective, all diseases that potentially influence joint space width were excluded (for example, signs of fracture, amputation, endocrinological diseases known to affect bone metabolism, rheumatic diseases, genetic diseases, oncological diseases, osteoarthritis).

#### Scoring of the radiographs

All radiographs were scored by the same two radiologists in a blinded manner using the Sharp Scores with the joint space narrowing as well as the erosion score segments [12], which evaluates joints of the hands as follows: Sharp Score for erosion, which evaluates 34 joints of the hands (total sum of points: 170); and Sharp Score for joint space narrowing, which evaluates 36 joints of the hands (total sum of points: 144). The scale is as follows: score 0 = normal joint; score 1 = initial reduction of the joint space width; score 2 = reduction of the joint space width <50%; score 3 = joint space narrowing >50%; and score 4 = ankylosis [12].

To reveal statistical evidence, the individual sum of scoring points was then divided by the number of evaluated joints and an average joint space narrowing score of the joints was obtained. If there was ambiguity in the blind assessment, a third radiologist reviewed the radiographs and provided the final decision.

### Ethics committee

All examinations were performed in accordance with the rules and regulations of the local Human Research and Ethics Committee of Friedrich-Schiller-University Jena. As a special note, the authors confirm that all radiographs used for CAJSA calculations were performed as part of routine clinical care; no additional radiographs were obtained only for study purposes.

### Statistical analysis

Statistical analysis was performed using SPSS^® ^Version 15.0 (SPSS, Chicago, IL, USA) for Windows.

#### Cross-sectional Z-Score analysis

The Spearman correlation coefficient was used to investigate associations between the Z-Score and the Sharp Score for joint space narrowing.

Severity-dependent joint space narrowing of the finger joints in the course of RA was evaluated based on the Sharp Score for joint space narrowing. The differences were calculated with the independent-sample *t *test.

All patients were divided in two groups with and without bone erosions to quantify sensitivity and specificity of the Z-Score as well as of the Sharp Score for joint space narrowing dependent on the occurrence of bone erosions. The sensitivity and specificity was evaluated by receiver operating characteristic curve analysis.

#### Longitudinal Z-Score analysis

The objective of the second study part was to quantify therapeutic changes of the Z-Score in RA patients undergoing therapy with methotrexate compared with leflunomide. The changes from first to second X-ray scans were compared within the groups by the Mann-Whitney *U *Test.

The significance level was considered with *P *< 0.05 as significant.

## Results

### Cross-sectional Z-Score analysis

#### Association between Z-Score and Sharp Score

The highest significant coefficient of correlation was observed between the Z-Score (MCP total) and the Sharp Score with *r *= 0.63 (*P *< 0.001). Regarding the PIP articulations, a lower significant correlation coefficient (*r *= 0.33; *P *< 0.01) of the Z-Score (PIP total) associated with the Sharp Score was revealed. Furthermore, the Z-Score of the DIP joints presented a nonsignificant coefficient of correlation compared with the Sharp Score (*r *= 0.15; *P *= not significant).

#### Reduction of finger joint space widths illustrated by the Z-Score dependent on disease severity

**Figure 2:**
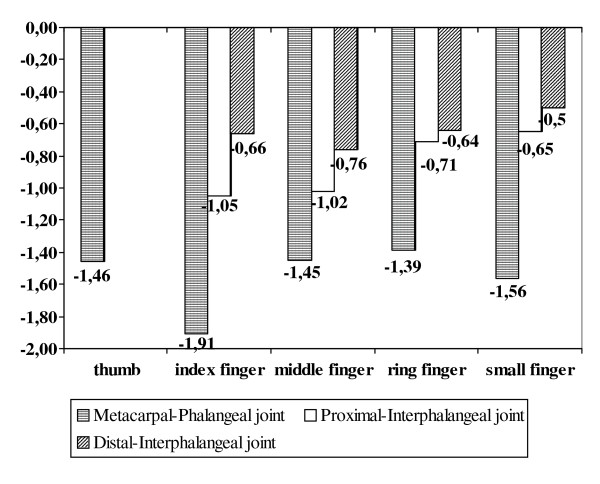
**Joint space width reduction based on computer-aided joint space analysis-specific Z-Scores**. Reduction of joint space widths based on the computer-aided joint space analysis-specific Z-Score differentiated for each finger and for all articulations between Sharp-van der Heijde score 0 and 3.

For MCP articulation (see Table 1 and Figure 2), the Z-Score (MCP total) showed a continuous significant decline (-1.65 ± 0.30 SDs) from 0.24 ± 0.33 SDs (score 0) to -1.41 ± 1.01 SDs (score 3). The index finger showed the strongest decrease in the Z-Score (-2.37 ± 0.43 SDs) from 0.46 ± 0.62 SDs (score 0) to -1.91 ± 1.57 SDs (score 3). The thumb and middle finger also presented a decrease of -1.74 ± 0.42 SDs (thumb) from 0.27 ± 0.62 SDs (score 0) to -1.46 ± 1.54 SDs (score 3) versus -1.77 ± 0.46 SDs (middle finger) from 0.32 ± 0.61 SDs (score 0) to -1.45 ± 1.69 SDs (score 3). The Z-Score also decreased for the ring finger by -1.39 ± 0.36 SDs and for the little finger by -1.56 ± 0.43 SDs.

**Table 1:** Finger joint space width reduction estimated by computer-aided joint space analysis for metacarpal-phalangeal articulation

	Z-Score					
	
Average Sharp Score for joint space narrowing	MCP (thumb)	MCP (index finger)	MCP (middle finger)	MCP (ring finger)	MCP (little finger)	MCP (total)
0	0.27 ± 0.62	0.46 ± 0.62	0.32 ± 0.61	0.21 ± 0.55	0.15 ± 0.58	0.24 ± 0.33
1	-0.31 ± 0.70	-0.37 ± 0.83	-0.11 ± 0.58	-0.05 ± 0.66	-0.11 ± 0.60	-0.16 ± 0.29
2	-0.39 ± 0.82	-0.70 ± 0.96	-0.39 ± 0.91	-0.11 ± 0.82	-0.33 ± 0.78	-0.39 ± 0.44
3	-1.46 ± 1.54	-1.91 ± 1.57	-1.45 ± 1.69	-1.18 ± 1.30	-1.41 ± 1.59	-1.41 ± 1.01
Difference between score 0 and 3	1.74 ± 0.42	2.37 ± 0.43	1.77 ± 0.46	1.39 ± 0.36	1.56 ± 0.43	1.65 ± 0.30
Significance, *P*	<0.01	<0.01	<0.01	<0.01	<0.01	<0.01

Data presented as mean ± standard deviation. Reduction of finger joint space widths from score 0 to 3 using the average Sharp-van der Heijde score as estimated by computer-aided joint space analysis for the metacarpal-phalangeal (MCP) articulation.

For PIP articulation (see Table 2 and Figure 2), the Z-Score (PIP total) significantly decreased (-0.96 ± 0.31 SDs) from 0.01 ± 0.46 SDs (score 0) to -0.95 ± 1.14 SDs (score 3). The index finger showed a significant decline (-1.23 ± 0.31 SDs) from 0.18 ± 0.84 SDs (score 0) to -1.05 ± 1.08 SDs (score 3). The Z-Score of the middle finger was significantly reduced by -1.07 ± 0.42 SDs from 0.05 ± 0.43 SDs (score 0) to -1.02 ± 0.39 SDs (score 3). The ring finger demonstrated a less significant reduction (-0.73 ± 0.35 SDs) from 0.02 ± 0.51 SDs (score 0) to -0.71 ± 1.27 SDs (score 3) as well as the little finger (-0.67 ± 0.29 SDs) from 0.02 ± 0.91 SDs (score 0) to -0.65 ± 1.40 SDs (score 3).

**Table 2:** Finger joint space width reduction estimated by computer-aided joint space analysis for proximal-interphalangeal articulation

	Z-Score				
	
Average Sharp Score for joint space narrowing	PIP (index finger)	PIP (middle finger)	PIP (ring finger)	PIP (little finger)	PIP (total)
0	0.18 ± 0.84	0.05 ± 0.43	0.02 ± 0.51	0.02 ± 0.91	0.01 ± 0.46
1	-0.34 ± 1.06	-0.25 ± 1.11	-0.11 ± 0.47	-0.28 ± 0.97	-0.27 ± 0.65
2	-0.47 ± 1.02	-0.43 ± 1.12	-0.41 ± 1.08	-0.35 ± 1.06	-0.42 ± 0.79
3	-1.05 ± 1.08	-1.02 ± 0.39	-0.71 ± 1.27	-0.65 ± 1.40	-0.95 ± 1.14
Difference between score 0 and 3	1.23 ± 0.31	1.07 ± 0.42	0.73 ± 0.35	0.67 ± 0.29	0.96 ± 0.31
Significance, *P*	<0.01	<0.05	<0.05	<0.05	<0.05

Data presented as mean ± standard deviation. Reduction of finger joint space widths from score 0 to 3 using the average Sharp-van der Heijde score as estimated by computer-aided joint space analysis for the proximal-interphalangeal (PIP) articulation.

For DIP articulation (see Table 3 and Figure 2), the Z-Score (DIP total) showed no significant decrease with -0.47 ± 0.34 SDs from -0.14 ± 1.02 SDs (score 0) to -0.61 ± 1.18 SDs (score 3). The index finger revealed a nonsignificant reduction of -0.65 ± 0.37 SDs from -0.01 ± 1.20 SDs (score 0) to -0.66 ± 1.28 SDs (score 3). The middle finger and ring finger presented a similar nonsignificant decrease between score 0 and score 3 of -0.58 ± 0.38 SDs versus -0.57 ± 0.47 SDs. The lowest decline was observed for the little finger, with -0.47 ± 0.47 SDs from -0.03 ± 0.97 SDs (score 0) to -0.50 ± 1.71 SDs (score 3).

**Table 3:** Finger joint space width reduction estimated by computer-aided joint space analysis for distal-interphalangeal articulation

	Z-Score				
	
Average Sharp Score for joint space narrowing	DIP (index finger)	DIP (middle finger)	DIP (ring finger)	DIP (little finger)	DIP (total)
0	-0.01 ± 1.20	-0.18 ± 1.00	-0.07 ± 1.54	-0.03 ± 0.97	-0.14 ± 1.02
1	-0.33 ± 0.71	-0.20 ± 1.30	-0.18 ± 0.72	-0.35 ± 0. 85	-0.22 ± 0.78
2	-0.53 ± 1.27	-0.34 ± 1.02	-0.37 ± 1.06	-0.41 ± 1.03	-0.39 ± 0.77
3	-0.66 ± 1.28	-0.76 ± 1.35	-0.64 ± 1.61	-0.50 ± 1.71	-0.61 ± 1.18
Difference between score 0 and 3	0.65 ± 0.37	0.58 ± 0.38	0.57 ± 0.47	0.47 ± 0.47	0.47 ± 0.34
Significance, *P*	NS	NS	NS	NS	NS

Data presented as mean ± standard deviation. Reduction of finger joint space widths from score 0 to 3 using the average Sharp-van der Heijde score as estimated by computer-aided joint space analysis for the distal-interphalangeal (DIP) articulation. NS, not significant.

Regarding sensitivity and specificity of the Z-Score and the Sharp Score for joint space narrowing dependent on the existence of erosions (see Table 4), for the Sharp Score for joint space narrowing a sensitivity and specificity of 48.3% and 100.0% (area under the curve 0.988, *P *< 0.01) were verified regarding the detection of erosions. A high sensitivity with a moderate specificity in the case of bone erosions was observed by the Z-Score, presenting the best data for MCP articulation of the index finger with 87.9% versus 55.2% (area under the curve 0.806, *P *< 0.01). The Z-Score (MCP total) revealed a sensitivity and specificity of 85.4% versus 55.2% (area under the curve 0.797, *P *< 0.01). Regarding the Z-Score of the PIP and DIP articulation (total), lower sensitivity of 67.5% (area under the curve 0.678, *P *< 0.01) and 53.8% (area under the curve 0.520, *P *= not significant) was observed.

**Table 4:** Z-Score sensitivity and specificity based on computer-aided joint space analysis dependent on onset of erosions

Z-Score	Sensitivity (%)	Specificity (%)
Metacarpal-phalangeal joint		
Thumb	73.2	51.7
Index finger	87.9	55.2
Middle finger	75.8	55.2
Ring finger	70.1	51.7
Little finger	77.1	51.7
Total	85.4	55.2
Proximal-interphalangeal joint		
Index finger	74.5	44.8
Middle finger	70.7	58.6
Ring finger	69.4	58.6
Little finger	63.1	51.7
Total	67.5	51.7
Distal-interphalangeal joint		
Index finger	58.6	41.4
Middle finger	53.5	48.3
Ring finger	62.4	41.4
Little finger	51.0	48.3
Total	53.5	37.9

### Longitudinal Z-Score analysis

#### Radiological progression

For both treatment groups (leflunomide and methotrexate), no significant changes of the Sharp Score for erosion and the Sharp Score for joint space narrowing over the observation period of 1.8 years were observed (see Table 5). The median Sharp Score for joint space narrowing of the first and second measurements was 1.

**Table 5:** Changes of Sharp Score for joint space narrowing and Z-Score over 1

	Sharp Score for joint space narrowing	Z-Score (standard deviations)
Leflunomide		
First X-ray scan	1	0.16
Second X-ray scan	1	0.13
Change	0	-0.03
Methotrexate		
First X-ray scan	1	0.08
Second X-ray scan	1	-0.05
Change	0	-0.13

Data presented as median.

#### Influence of disease-modifying antirheumatic drug therapy on the Z-Score

For the methotrexate-treated patients, the Z-Score (MCP total) decreased (-0.13 SDs) from 0.08 SDs (initial measurement) to -0.05 SDs (second measurement) (see Table 5). Regarding the leflunomide-treated group, the Z-Score (MCP total) was not significantly reduced (-0.03 SDs) from 0.16 SDs (initial measurement) to 0.13 SDs (second measurement).

## Discussion

CAJSA based on digital radiographs clearly offers a superior quantification of joint space narrowing in RA. The aim of this study was to elucidate the value of the Z-Score for the RA-related quantification of finger joint space narrowing depending on the severity of RA as well as the sensitivity and specificity in dependence on visible bone erosions. Furthermore, the clinical relevance of the Z-Score was determined in the comparison of two different patient groups treated with disease-modifying antirheumatic drugs (methotrexate and leflunomide).

### Technical implementation of the computer-aided joint space analysis technique

A previous study showed that technical parameters, such as exposure level, film brand, film sensitivity, and film focus distance, do not affect the reproducibility of CAJSA measurements during the image-acquisition process [13]. Recently published data presented no influence of hand rotation on CAJSA measurements with the exception of a hand rotation of more than 15° during X-ray imaging, which is complementary to an oblique acquired hand radiograph and should be not used for CAJSA measurements [11]. Concerning reproducibility, measurements of joint space widths by CAJSA are reliable due to the high inter-radiograph reproducibility (CAJSA measurements were evaluated on 10 radiographs of the same subject that were performed with repositioning under standard X-ray settings) for conventional hand radiographs (coefficient of variation 0.66%) and digital hand radiographs (coefficient of variation 0.63%). Additionally, an excellent intra-radiograph reproducibility (10 repeated CAJSA analyses of the same hand radiograph) revealed an advanced coefficient of variation with 0.54% for conventional and 0.38% for digital imaging techniques [11]. Furthermore, CAJSA measurements implied a decrease of reproducibility between Sharp-van der Heijde Scoring method score 0 (coefficient of variation 0.37%) and Sharp-van der Heijde Scoring method score 3 (coefficient of variation 1.37%) based on the more complicated contour finding of joint margins in higher grades of RA joint destruction [11].

### Influence on finger joint space width

The influence of body size and influence of body weight are potential factors that could influence the measurement of finger joint space width. In this context, data for this observation are very rare. A cross-sectional study of RA patients revealed no influence of body mass index on CAJSA measurements for the MCP, PIP and DIP articulations, excluding MCP JSD in underweight RA patients with body mass index <20 kg/m^2 ^[8]. Generally, there is no significant influence of body weight, height, and body mass index on CAJSA measurements.

Recently published studies have evaluated finger joint space widths in healthy Caucasians as estimated by CAJSA. These results revealed a continuous reduction of finger joint space widths dependent on age and gender. Women demonstrated a significantly smaller joint space width (MCP JSD = -11.1%, PIP JSD = -15.4% and DIP JSD = -16.7%) compared with men. In healthy subjects, joint space widths also showed a significant reduction between age 30 and 79 years (MCP JSD = -20.1%, PIP JSD = -21.4% and DIP JSD = -24.8%) [7,14,15]. Goligher and colleagues estimated JSD at the MCP articulation in patients with early RA by a computerized, semi-automated joint space width analysis. The authors also found a narrowing of MCP JSD (-7.2%; not significant) between patients aged under 50 years compared with those over 60 years [16]. In a previous trial, Pfeil and colleagues confirmed a significant age-dependent decrease (age 20 to 39 years compared with age 60 to 79 years) of the MCP JSD (-24.8%) in RA patients. Additionally, their study showed an expected narrowing of joint space widths in women (JSD MCP = -10.4%, PIP JSD = -11.7% and DIP JSD = -16.0%) compared with men [8].

### Z-Score for quantification of finger joint space narrowing

Reliable differentiation between RA-related versus age-dependent and gender-dependent joint space narrowing is very difficult, highlighting the need to implement normative data. A possible solution is the use of an age-independent and gender-independent parameter for the quantification of JSD. The Z-Score of the peripheral finger articulations and their joint space widths offers the advantage for an age-independent and gender-independent quantification of joint space alterations in RA.

The study presented a highly significant correlation between the Z-Score of the MCP articulations and the Sharp Score for joint space narrowing. Our data demonstrated a continuously significant joint space narrowing as measured by the Z-Score using the Sharp Score for joint space narrowing for assessment of RA severity. The Z-Score (MCP total) showed a continuous significant decline from 0.24 ± 0.33 SDs (Sharp Score for joint space narrowing = 0) to -1.41 ± 1.01 SDs (Sharp Score for joint space narrowing = 3). Regarding the PIP articulations, the Z-Score (PIP total) significantly decreased from 0.02 ± 0.46 SDs (Sharp Score for joint space narrowing = 0) to -0.95 ± 1.14 SDs (Sharp Score for joint space narrowing = 3). The Z-Score of the DIP articulations (total) showed no significant results. Regarding the DIP joints, a nonsignificant correlation was observed between the Z-Score and the Sharp Score for joint space narrowing. The Z-Score was able to show early manifestations of RA at the MCP articulations [17] and to quantify joint destruction (also indicated by the Sharp Score for joint space narrowing). Furthermore, the lack of correlation between the Z-Score of the DIP joints and the Sharp Score for joint space narrowing was expected and also in accordance with the non-involvement of DIP joints in RA. In the case of a reduced Z-Score of the MCP and PIP articulations with an absence of joint space reduction of the DIP joints, the normal joint space widths of DIP joints can be used as a diagnostic criterion for RA.

Furthermore, the study by Pfeil and colleagues observed positive Z-Scores (MCP articulations) for a Sharp Score for joint space narrowing of 0 (1.86 ± 0.15 SDs) [9]. This study confirmed positive Z-Scores for the MCP (0.24 ± 0.33 SDs) and PIP articulations (0.02 ± 0.46 SDs) based on a Sharp Score for joint space narrowing of 0. A Sharp Score for joint space narrowing of 0 is defined as a joint space width with an absence of joint space narrowing. The positive Z-Scores for a Sharp Score for joint space narrowing of 0 indicate that these RA patients have a larger joint space than healthy subjects. This phenomenon is caused by joint effusion and disease-related synovitis in the early stages of RA, followed by a joint space narrowing in the prolonged course of RA [9,18].

Our observations also revealed an advanced narrowing of the Z-Score for the MCP articulations from a Sharp Score for joint space narrowing of 2 (-0.39 ± 0.44 SDs) to a Sharp Score for joint space narrowing of 3 (-1.41 ± 1.01 SDs). Using the Sharp Score for joint space narrowing, the Z-Score of the MCP articulations confirmed an accentuated reduction from score 2 (-0.39 ± 0.57 SDs) to score 3 (-1.83 ± 1.28 SDs) [9]. This result could be explained by the advanced difference of joint destruction in RA patients between score 3 (reduction of the joint space width >50%) compared with score 2 (reduction of the joint space width <50%) quantified by the Sharp Score for joint space narrowing, respectively.

### Z-Score for evaluation of bone erosions and as a surrogate marker of RA progression

The general consensus is that inflammation leads to structural damage including joint space narrowing and periarticular erosions in RA [19]. Based on the data of the ASPIRE trial, the evaluation of RA progression based on erosions and joint space narrowing as a parallel or independent process was performed [19,20]. On the one hand, the study clearly verified that worsening of erosions leads to progression of erosions and the worsening of joint space narrowing predisposes to progression of joint space narrowing in early RA [19,20]. On the other hand, the ASPIRE trial points out that joint space narrowing at baseline is associated in 9.5% with the formation of erosion and in 3.5% with the worsening of joint space narrowing [20]. Consecutively, an interesting question is the association between joint space narrowing as measured by the Z-Score and the onset of bone erosions. Our data revealed a high sensitivity (85.4%) for MCP JSD (total) in the case of visible bone erosions. For the conventional Sharp Score for joint space narrowing, a sensitivity and specificity of 48.3% and 100.0% was verified. The results showed that the prediction of erosions by the Sharp Score for joint space narrowing is low. These facts points out the predictive value of the Z-Score in the identification of erosive RA courses. Additionally a normal joint space width as quantified by the Sharp Score for joint space narrowing is not associated with erosions.

### Clinical relevance of the Z-Score

The treatment with disease-modifying antirheumatic drugs could diminish the progression of erosions and joint space narrowing. The Z-Score was able to differentiate between different treatment groups in RA.

The first study to our knowledge that evaluated the CAJSA-based joint space measurement technique for value as a therapy control tool was initiated by Pfeil and coworkers [21]. In this initial retrospective pilot study with 40 patients, a different therapeutic potency between methotrexate and leflunomide showed a remarkable reduced joint space narrowing for individuals treated with leflunomide [21]. This advanced multicenter study including 94 patients based on the LEMERADIX REGISTER revealed no significant changes of the Sharp Score for joint space narrowing in head-to-head comparison with the Z-Score. An accentuated stabilization of joint space narrowing could be identified for the Z-Score of the MCP joints in both subgroups treated with leflunomide and methotrexate, and the Z-Score was able to quantify therapeutic effects in a longitudinal study design. In a longitudinal study, Sharp and colleagues demonstrated a reduced structural damage in RA patients under leflunomide therapy; both study cohorts (MN302 und US301) showed increased values of up to 1.48 for leflunomide and up to 1.08 for methotrexate estimated by means of the Sharp Score for joint space narrowing [22]. Taking into consideration the radiogeometric assessment of RA progression, the results from the study conducted by van der Heijde and colleagues in 128 patients with a mean leflunomide treatment duration of 4.3 years demonstrated no radiographically visible progression in 33% of the RA patients during the leflunomide therapy [23]. Larsen and colleagues radiographically demonstrated a delay in disease progression under leflunomide application compared with sulfasalazine, which was observed as early as 6 months after the start of treatment and was still effective after 24 months of treatment [24]. Further prospective therapy studies are necessary to confirm the value of the Z-Score in the evaluation of therapeutic effects in patients with early as well as prolonged RA.

A study comparing hand disability assessed by questionnaire and joint damage measured by the Sharp Scores for erosion and for joint space narrowing has been undertaken by Smolen and colleagues. The study revealed that an increasing Sharp Score for erosion did not lead to an increasingly irreversible hand disability, but an increasing Sharp Score for joint space narrowing is significantly associated with more severe hand disability [25]. This finding indicates that irreversible hand disability may be primarily mediated by cartilage destruction and not by bone damage due to bone erosion. Further studies using the CAJSA technique should be performed to illustrate these important results.

A potential limitation of the CAJSA technique is the impossible usage in patients with a Sharp Score for joint space narrowing of 4, which is characterized by ankylosis, subluxation, and luxation. Especially, three-dimensional deformities such as subluxation and luxation are potential evaluation errors for the CAJSA technique. On the contrary, the CAJSA system includes integrated self-checking that detects a malalignment of the bone edges and automatically interrupts the measurement process. Furthermore, the study of Pfeil and colleagues revealed no influence of hand rotation on CAJSA measurements using plain radiographs in anterior-posterior projection [11]. Otherwise, modern therapeutic strategies will hopefully limit the number of these patients with advanced joint destruction and the CAJSA technique can also be used in the diagnosis of early RA.

## Conclusion

This study presents the severity-dependent reduction of joint space widths using the Z-Score based on CAJSA estimates in patients with RA, in particular the MCP JSD of the second and third fingers as a surrogate marker of RA progression. The Z-Scoring of the CAJSA method would also help to identify those patients with aggressive RA who develop joint damage before visible erosions occur and enables a reliable as well as more precise estimation of joint space widths without influence of age or gender. Additionally, stabilization of the joint space narrowing was observed for patients treated with disease-modifying antirheumatic drugs. This technique is also cost-effective, allows a timely diagnosis of RA, whether in early or later disease stages, and potentially provides an earlier planning of appropriate therapeutic strategies.

## Abbreviations

CAJSA: computer-aided joint space analysis; DIP: distal-interphalangeal; JSD: joint space distance; MCP: metacarpal-phalangeal; PIP: proximal-interphalangeal; RA: rheumatoid arthritis; SD: standard deviation.

## Competing interests

The authors declare that they have no competing interests. JB and AP have received speaker's fees from Sanofi-Aventis. KB is a full-time employee of Sanofi-Aventis Deutschland GmbH, Berlin, Germany. The longitudinal study was funded and managed by Sanofi-Aventis Deutschland GmbH (LEFLU_L_04198).

## Authors' contributions

AP, JB, KB, PO and GW contributed to the study design. AP, JB, KB and PO organized the data collection. AP, JB and AH read and scored the hand radiographs. AP, DMR and KB contributed the statistical analysis. AP, DMR and GL performed the literature search. AP, JB, PO, KB and GW contributed to data interpretation and manuscript preparation. DMR and GL edited the manuscript. All authors read and approved the manuscript for publication.

## References

[B1] Bridges SL Jr Causey ZL Burgos PI Huynh BQ Hughes LB Danila MI van Everdingen A Ledbetter S Conn Dl Tambane A Westfall AO Jonas BL Callahan LF Smith ES Brasington R Moreland LW Alarcón GS van der Heijde D Radiographic severity of rheumatoid arthritis in African Americans: results from a multicenter observational study Arthritis Care Res (Hoboken) 2010 62 624 631 10.1002/acr.20040 20461784 PMC3052790

[B2] Lukas C Sharp JT Angwin J Boers M Duryea J Hall JR Kaufman JA Landewé R Langs G Lukas C Maillefert JF Bernelot Moens HJ Peloschek P Strand V van der Heijde D Automated measurement of joint space width in small joints of patients with rheumatoid arthritis J Rheumatol 2008 35 1288 1293 18597408

[B3] Pfeil A Haugeberg G Hansch A Renz DM Lehmann G Malich A Wolf G Böttcher J The value of digital X-ray radiogrammetry in the assessment of inflammatory bone loss in rheumatoid arthritis Arthritis Care Research (Hoboken) 2011 63 666 674 10.1002/acr.20423 21557522

[B4] Böttcher J Pfeil A Rosholm A Petrovitch A Seidl BE Malich A Schäfer ML Kramer A Mentzel HJ Lehmann G Hein G Kaiser WA Digital X-ray radiogrammetry combined with semi-automated analysis of joint space distances as a new diagnostic approach in rheumatoid arthritis - a cross-sectional and longitudinal study Arthritis Rheum 2005 52 3850 3859 10.1002/art.21606 16320332

[B5] Sharp JT Angwin J Boers M Duryea J von Ingersleben G Hall JR Kauffman JA Landewé R Langs G Lukas C Maillefert JF Bernelot Moens HJ Peloschek P Strand V van der Heijde D Computer based methods for measurement of joint space width: update of an ongoing OMERACT project J Rheumatol 2007 34 874 883 17407243

[B6] Neumann G Depablo P Finckh A Chibnik LB Wolfe F Duryea J Patient repositioning reproducibility of joint space width (JSW) measurements on hand radiographs Arthritis Care Res (Hoboken) 2011 63 203 207 10.1002/acr.20374 20957661 PMC3031752

[B7] Pfeil A Böttcher J Seidl BE Heyne JP Petrovitch A Eidner T Mentzel HJ Wolf G Hein G Kaiser WA Computer-aided joint space analysis of the metacarpal-phalangeal and proximal-interphalangeal finger joint - normative age-related and gender specific data Skeletal Radiol 2007 36 853 864 10.1007/s00256-007-0304-8 17508211

[B8] Pfeil A Hansch A Lehmann G Eidner T Schäfer ML Oelzner P Renz DM Wolf G Hein G Kaiser WA Böttcher J Impact of sex, age, body mass index and handedness on finger joint space width in patients with prolonged rheumatoid arthritis using computer-aided joint space analysis Rheumatol Int 2009 29 517 524 10.1007/s00296-008-0728-z 18953542

[B9] Pfeil A Schäfer ML Lehmann G Seidl BE Eidner T Malich A Renz DM Oelzner P Hansch A Wolf G Hein G Kaiser WA Böttcher J Implementation of Z-Scores as an age- and gender-independent parameter for estimating joint space widths in rheumatoid arthritis J Rheumatol 2009 36 717 723 10.3899/jrheum.080651 19286851

[B10] Arnett FC Edworthy SM Bloch DA McShane DJ Fies FJ Cooper NS The American Rheumatism Association 1987 revised criteria for the classification of rheumatoid arthritis Arthritis Rheum 1988 31 315 324 10.1002/art.1780310302 3358796

[B11] Pfeil A Sommerfeld J Hansch A Fröber R Renz DM Lehmann G Malich A Wolf G Böttcher J Reproducibility and influence of hand rotation on computer-aided joint space analysis Joint Bone Spine 2012 79 384 388 10.1016/j.jbspin.2011.07.011 21963809

[B12] Sharp JT Young DY Bluhm GB Brook A Brower AC Corbett M Decker JL Genant HK Gofton JP Goodsman N Larsen A Lidski MD Pussila P Weinstein AS Weissman BN How many joints in the hands and wrists should be included in a score of radiologic abnormalities used to assess rheumatoid arthritis? Arthritis Rheum 1985 28 1326 1335 10.1002/art.1780281203 4084327

[B13] Pfeil A Sommerfeld J Fröber R Lehmann G Malich A Hansch A Wolf G Böttcher J Feasibility study of semi-automated measurements of finger joint space widths Rheumatol Int 2011 31 1349 1354 10.1007/s00296-010-1468-4 20401484

[B14] Pfeil A Böttcher J Schäfer ML Seidl BE Schmidt M Petrovitch A Heyne JP Lehmann G Oelzner P Hein G Wolf G Kaiser WA Normative reference values of joint space width estimated by computer-aided joint space analysis (CAJSA): the distal-interphalangeal joint J Digit Imaging 2008 21 Suppl 1 104 112 17384977 10.1007/s10278-007-9031-x PMC3043877

[B15] Pfeil A Böttcher J Seidl BE Schäfer ML Hansch A Heyne JP Petrovitch A Mentzel HJ Eidner T Wolf G Hein G Kaiser WA Computer-aided joint space analysis (CAJSA) of the proximal-interphalangeal joint - normative age-related and gender specific data Acad Radiol 2007 14 594 602 10.1016/j.acra.2007.01.032 17434073

[B16] Goligher EC Duryea J Liang MH Finckh A Radiographic joint space width in the fingers of patients with rheumatoid arthritis of less than one year's duration Arthritis Rheum 2006 54 1440 1443 10.1002/art.21829 16645973

[B17] van der Heijde DM How to read radiographs according to the Sharp/van der Heijde method J Rheum 1999 26 743 745 10090194

[B18] Rau R Wassenberg S Scoringmethoden bei der rheumatoiden Arthritis. Rau R. Deutsche Gesellschaft für Rheumatolgie, Kommission bildgebende Verfahren. Bildgebende Verfahren in der Rheumatologie Steinkopf Verlag, Darmstadt 2007 S27

[B19] van der Heijde DM Erosions versus joint space narrowing in rheumatoid arthritis. What do we know? Ann Rheum Dis 2011 Suppl 1 116 118 10.1136/ard.2010.140525 21339214

[B20] Smolen JS van der Heijde DM Aletaha D Xu S Han J Baker D St Clair EW Progression of radiographic joint damage in rheumatoid arthritis: independence of erosions and joint space narrowing Ann Rheum Dis 2009 68 1535 1540 10.1136/ard.2008.094128 18957487

[B21] Pfeil A Lippold J Eidner T Lehmann G Oelzner P Renz DM Hansch A Wolf G Hein G Kaiser WA Böttcher J Effects of leflunomide and methotrexate in rheumatoid arthritis detected by digital X-ray radiogrammetry and computer-aided joint space analysis Rheumatol Int 2009 29 287 295 10.1007/s00296-008-0682-9 18787830

[B22] Sharp JT Strand V Leung H Hurley F Loew-Friedrich I Treatment with leflunomide slows radiographic progression of rheumatoid arthritis. Results from three randomized controlled trials of leflunomide in patients with active rheumatoid arthritis Arthritis Rheum 2000 43 495 505 10.1002/1529-0131(200003)43:3<495::AID-ANR4>3.0.CO;2-U 10728741

[B23] van der Heijde D Kalden J Scott D Smolen J Strand V Long term evaluation of radiographic disease progression in a subset of patients with rheumatoid arthritis treated with leflunomide beyond 2 years Ann Rheum Dis 2004 63 737 739 10.1136/ard.2003.010983 15140783 PMC1755041

[B24] Larsen A Kvien TK Schattenkirchner M Rau R Scott DL Smolen JS Rozman B Westhovens R Tikly M Oed C Rosenburg R European Leflunomide Study Group Slowing of disease progression in rheumatoid arthritis patients during long-term treatment with leflunomide or sulfasalazine Scand J Rheumatol 2001 30 135 142 10.1080/030097401300162897 11469522

[B25] Smolen JS Funovits J Aletaha D Irreversible physical disability in rheumatoid arthritis is determined by cartilage damage rather than bone destruction Ann Rheum Dis 2010 69 Suppl 3 655 10.1136/ard.2010.138693 21321002

